# The epigenetic integrator UHRF1: on the road to become a universal biomarker for cancer

**DOI:** 10.18632/oncotarget.17393

**Published:** 2017-04-24

**Authors:** Waseem Ashraf, Abdulkhaleg Ibrahim, Mahmoud Alhosin, Liliyana Zaayter, Khalid Ouararhni, Christophe Papin, Tanveer Ahmad, Ali Hamiche, Yves Mély, Christian Bronner, Marc Mousli

**Affiliations:** ^1^ Laboratory of Biophotonics and Pharmacology, Faculty of Pharmacy, University of Strasbourg, Illkirch, France; ^2^ Institute of Genetics and Molecular and Cellular Biology, University of Strasbourg, Illkirch-Graffenstaden, France; ^3^ Department of Biochemistry, Faculty of Sciences, King Abdulaziz University, Jeddah, Saudi Arabia; ^4^ Cancer Metabolism and Epigenetic Unit, King Abdulaziz University, Jeddah, Saudi Arabia; ^5^ Cancer and Mutagenesis Unit, King Fahd Centre for Medical Research, King Abdulaziz University, Jeddah, Saudi Arabia

**Keywords:** cancer, biomarkers, epigenetics, UHRF1, DNA methylation

## Abstract

Cancer is one of the deadliest diseases in the world causing record number of mortalities in both developed and undeveloped countries. Despite a lot of advances and breakthroughs in the field of oncology still, it is very hard to diagnose and treat the cancers at early stages. Here in this review we analyze the potential of Ubiquitin-like containing PHD and Ring Finger domain 1 (UHRF1) as a universal biomarker for cancers. UHRF1 is an important epigenetic regulator maintaining DNA methylation and histone code in the cell. It is highly expressed in a variety of cancers and is a well-known oncogene that can disrupt the epigenetic code and override the senescence machinery. Many studies have validated UHRF1 as a powerful diagnostic and prognostic tool to differentially diagnose cancer, predict the therapeutic response and assess the risk of tumor progression and recurrence. Highly sensitive, non-invasive and cost effective approaches are therefore needed to assess the level of UHRF1 in patients, which can be deployed in diagnostic laboratories to detect cancer and monitor disease progression.

## INTRODUCTION

In cancer, the prognosis of the disease is highly dependent on the type and location of the cancer along with the stage at which it is diagnosed. The survival rate and the treatment response is better if the cancer is diagnosed early when the tumor is localized and small. Nowadays many biomolecules and epigenetic patterns are being explored as “biomarkers” to help in early diagnosis of cancers along with currently employed techniques of imaging and cytology [1]. An ideal biomarker for cancer detection must be able to differentiate between normal and tumoral cells and it should be able to predict the malignant potential and prognosis of the disease.

All cells of a multicellular mammalian organism, except germinal cells, contain the same DNA in terms of nucleotide sequence. Considering the fact that DNA is the layer of heredity and cell identity, how can cell diversity and differentiation arise from the same DNA sequence is an important question challenging the scientific community. Epigenetics is the research field that tries to answer this question by deciphering a tremendous number of cellular mechanisms of gene regulation embedded in the chromatin but not related to changes in DNA sequences. In other words, it refers to external modifications of DNA that turn *genes* “on” or “off. At the molecular level, “off” means that the genes are silenced, by means of DNA methylation and histone methylation, *e.g*., di- and tri- methylation of lysines 9 & 27 of histone H3 (H3K9me2, H3K9me3, H3K27me2, H3K27me3) as well as chromatin structure, micro RNA and histone variants [2–5]. However, gene expression does not function as a simple “on-off” dichotomy but rather through a complex language dictated by the degree of DNA methylation and a set of epigenetic marks appearing on the N-terminal tails of histones present in the nucleosome [3]. This complex language allows the cell to express genes as a function of precise needs during cell cycle or during lifespan and no more or less than it is required for the cell to work adequately. This complex language is profoundly modified in various diseases, including cancer [3–5].

Indeed, cancer cells exhibit profound changes in epigenetic profiles, as much on the DNA methylation side as on histone code side [6]. Cancer cells undergo global DNA hypomethylation, whereas some regions, on the contrary, undergo hypermethylation, *e.g.* promoters of tumor suppressor genes [7, 8]. On the histone code versant, several modifications have been reported in various types of cancer [9].

There are increased evidences that DNA methylation appears as an ideal biomarker for various types of cancers [10–13]. DNA methylation in mammals preferentially occurs in a CpG context, meaning that both DNA strands are methylated in an asymmetrical manner, which represents one of the layers of epigenetic information. Methylation of cytosine is slightly mutagenic, explaining the loss of CpG sites in mammalian genomes during evolution. As a consequence, CpG sites in human genome are globally found 3–4 times less often than statistically expected, except in CpG islands, which are often located in gene promoters [2, 14].

The mechanism of inheritance of the methylation patterns is relatively well documented regarding DNA but is still elusive concerning histones, although several models are under investigation for definitive validation [15]. Duplication of DNA methylation patterns in a CpG context, is subjected to prior DNA replication generating hemi-methylated DNA, *i.e*., only one DNA strand is methylated, a state that is specifically recognized by Ubiquitin-like containing PHD and Ring Finger domain protein 1 (UHRF1) [16–20]. The sensing of hemi-methylated DNA by UHRF1, induces the recruitment of DNA methyltransferase 1 (DNMT1) which methylates the opposite unmethylated DNA strand, and consequently CpG dinucleotides are methylated on both strands. Through these properties, the tandem UHRF1/DNMT1 plays a role during cell proliferation and therefore in development and cancer [21].

## THE EPIGENETIC INTEGRATOR UHRF1

### Structure of UHRF1

Among the different epigenetic modulators, UHRF1, which is also known as Inverted CCAAT box Binding Protein of 90 kDa (ICBP90) or nuclear protein of 95kDa (Np95) [22–24] has gained a considerable attention during the past few years because of its high expression in most of the cancers and its ability to link important epigenetic processes such as DNA methylation and histone modifications [25].

**Figure 1:**
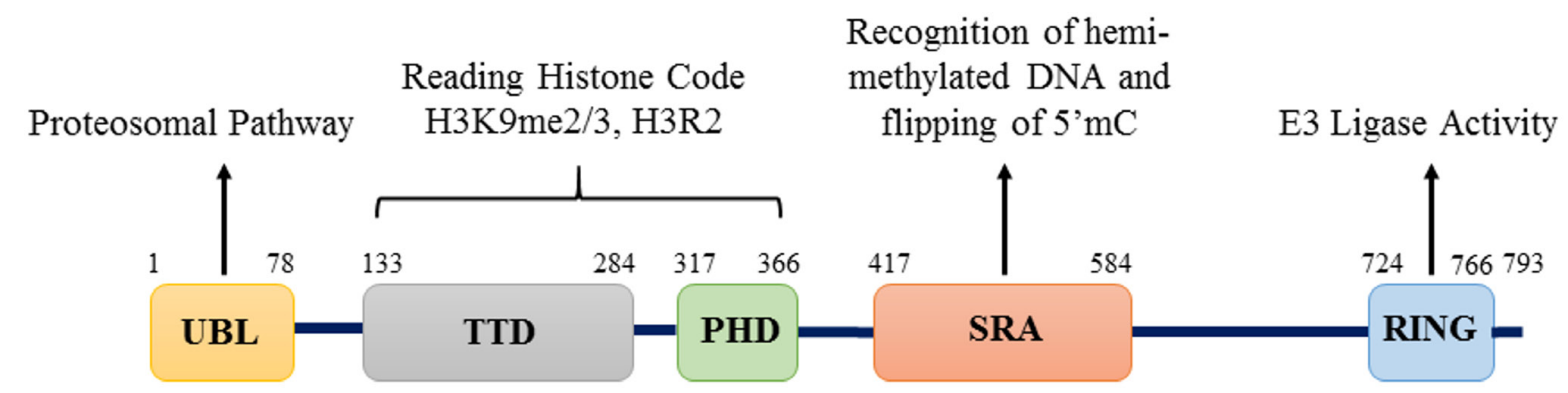
Structure of UHRF1 protein Structure of UHRF1 protein showing the different domains and their functions. The protein contains 793 amino acids and five major domains: UBL (ubiquitin-like) domain, TTD (Tandem Tudor Domain), PHD (Plant Homeodomain), SRA (Set and Ring Associated) domain and RING (Really Interesting New Gene) domain.

Initially, UHRF1 was identified as a transcription factor regulating the expression of topoisomerase IIα by binding to an inverted CCAAT box located in its promoter [22]. UHRF1 was further shown to critically participate in various epigenetic processes by its different structural domains (Figure 1). Indeed, UHRF1 is composed of an N-terminal ubiquitin-like domain that is coming before the tandem tudor domain (TTD) and plant homeodomain (PHD). These domains are followed by the unique set and ring associated (SRA) domain and the really interesting new gene (RING) finger domain at the C-terminus [25]. Except for the RING domain exhibiting an E3 ligase activity towards histone H3 on lysine 23 or on lysine 18, no further enzymatic activity has been so far identified for any of the other domains. Instead, interesting binding activities were identified for each domain conferring unique capacities of readout [26–28]. One key property of UHRF1 is its ability to sense the presence of hemi-methylated DNA at the replication fork, thanks to the SRA domain [19, 20]. Concomitantly, it can also sense the di- and tri- methylated lysine 9 of histone H3 (H3K9me2/H3K9me3) in the chromatin by help of its tandem tudor domain [29–31]. Association of UHRF1 with methylated H3K9 through TTD facilitates the maintenance of DNA methylation but primarily it is the SRA domain that recruits UHRF1 to hemi-methylated DNA [32]. Indeed, we have shown that the binding of SRA domain does not induce distortion of the DNA, which is in favor of a sliding behavior along the DNA seeking for hemi-methylated CpG sites and subsequent flipping of the methylated cytosine, thus facilitating the recruitment of DNMT1 [33, 34]. It has also been shown that UHRF1, through its SRA domain, is capable of recognizing hydroxymethylcytosine [35]. The relevance of this latter remains elusive but it might bring new insights in DNA methylation maintenance, once resolved.

Beside this role, UHRF1 is considered to play a pivotal role in the epigenetic inheritance as it coordinates the action of different chromatin modifying proteins [36]. It interacts, among many others, with DNA methyltransferases (DNMTs), proliferating cell nuclear antigen (PCNA), histone deacetylase 1 (HDAC1), ubiquitin specific protease 7 (USP7), euchromatic histone-lysine N methyltransferase 2 (G9a/EHMT2) and Tat Interacting Protein 60 (Tip60) to maintain DNA methylation patterns and histone epigenetic marks in various physiological and pathological conditions [18, 19, 37–42]. Together with its partners, UHRF1 ensures the regulation, through “silencing” of a high number of tumor suppressor genes and long non-coding RNAs, including *RB1* [43], *p16* (*CDKN2A)* [44–48], *CDH13* and *SHP1* [49], *SOCS3* and *3OST2* [50], *BRCA1* [51], *CDX2, RUNX3, FOXO4, PPARG* and *PML* [52, 53], *MEG3* [54] and *14-3-3σ* [55]. Moreover, *KISS1*, functioning as a metastasis suppressor in various cancers, also looks to be under the control of UHRF1 [56]. Altogether, these studies highlight UHRF1 as a conductor of tumor suppressor gene silencing in cancers through a DNA methylation-dependent mechanism.

### UHRF1 as a tumor promoter

UHRF1 is mostly expressed in proliferating cells, while it is not found in fully differentiated tissues [22]. Levels of UHRF1 expression positively co-relate with the proliferative potential of cells. In cancer cells, UHRF1 is overexpressed and promotes the proliferation and dedifferentiation of cells [22]. In non-cancerous proliferating cells, UHRF1 expression is cell cycle regulated and peaks in late G1 and G2/M phase, while in cancerous cells, UHRF1 is continuously expressed at all stages of cell cycle [57]. UHRF1 is considered to be essential for G1/S phase transition as its depletion or down-regulation by activation of p53/p21^Cip1/WAF1^ dependent DNA damage response leads to cell cycle arrest at the G1/S phase transition [58, 59]. Similarly, in another study it has been reported that depletion of UHRF1 in HCT116 cells leads to the activation of DNA damage response with subsequent cell cycle arrest at G2/M phase and induction of caspase 8-dependent apoptosis [60]. Conversely, overexpression of UHRF1 in human fibroblasts or its orthologue Np95 in terminally differentiated mouse myotubes facilitates the entry of these cells in S-phase and induces cell proliferation [43, 58]. The possibility that UHRF1 behaves as an oncogene has been questioned for a while [61]. However, it is now clearly demonstrated through a recent series of studies that UHRF1 is a tumor promoter. Indeed, it was shown that overexpressed UHRF1 causes DNA hypomethylation, a hallmark of cancer cells; instead of normal maintenance of DNA methylation. Overexpressed UHRF1, through its E3 ligase activity, ubiquitinylates DNMT1 and DNMT3. Thus, by destabilizing and delocalizing them, UHRF1 induces global DNA hypomethylation [62, 63].

Several studies have also revealed that disruption of UHRF1 function results in hypersensitivity to DNA damage [64–69] supporting the idea that UHRF1 plays a critical role in the maintenance of genome stability. This is not surprising, considering that a native protein has first a physiological role before a deleterious role. The deleterious role is coming from an abnormal level of UHRF1 rather than from its function itself.

**Figure 2:**
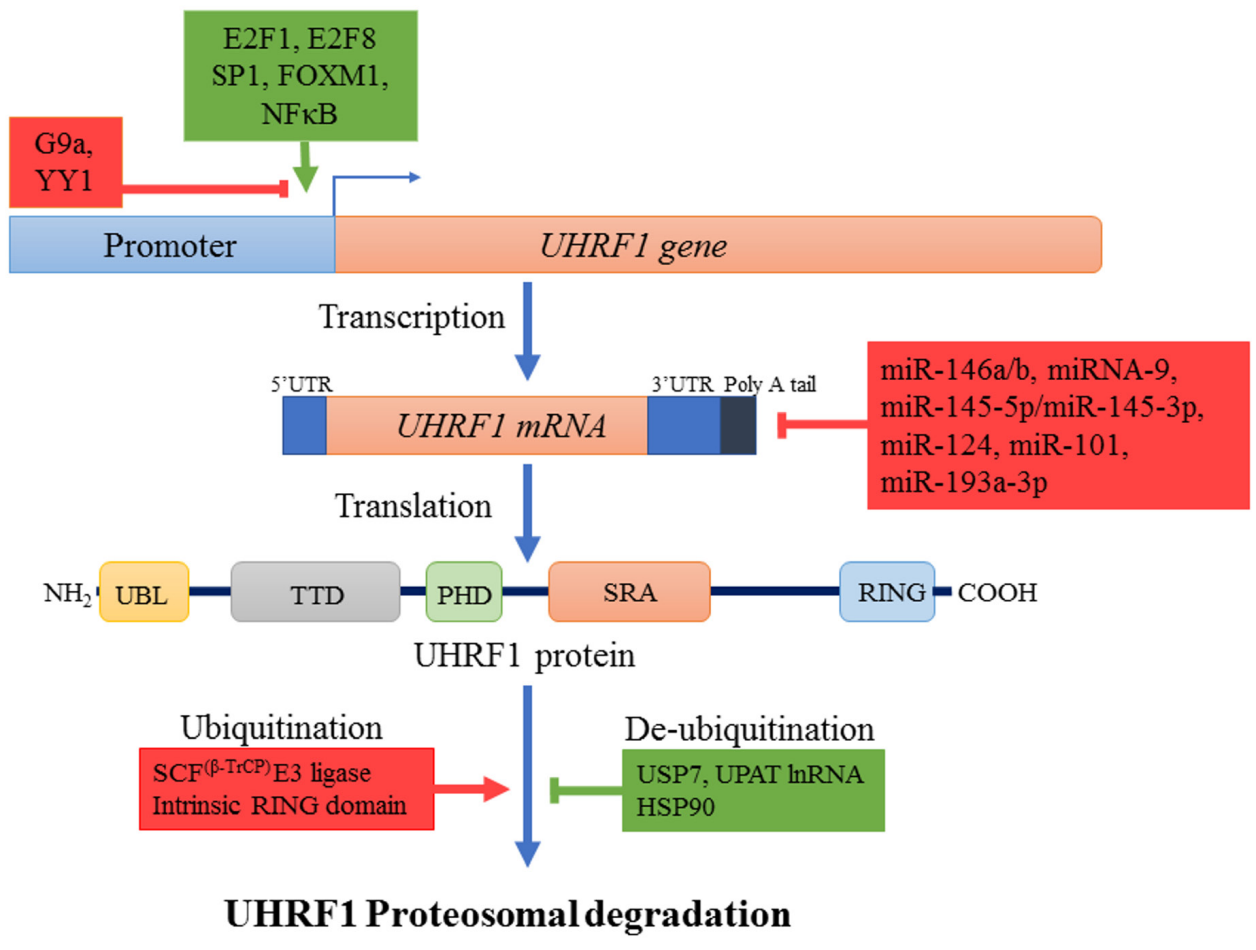
Regulation mechanisms of UHRF1 Different transcription factors like E2F1, E2F8, Sp1, FOXM1, NFκB (indicated in green) enhance while others such as YY1 along with lysine methyl transferase G9a (indicated in red) repress the expression of *UHRF1* at transcription level. Many small non-coding microRNAs also decrease *UHRF1* expression by destabilizing *UHRF1 mRNA* through binding to 3’UTR region. UHRF1 protein is degraded by proteosomal pathway after autoubiquitinylation or ubiquitinylation by SCF^β-TrCP^ E3 ligase. Ubiquitinylated UHRF1 is stabilized in cells by USP7, HSP90 or UPAT lnRNA. Increased transcription factor expression, downregulation of miRNAs and increased levels of stabilizing factors (all indicated in green) result in overexpression of UHRF1.

The abnormally high level of UHRF1 may result from the aberrant activity of various transcription factors regulating the expression of UHRF1 in cancers (Figure 2). E2F transcription factor 1 (E2F1) and E2F transcription factor 8 (E2F8) are upregulated in many cancers and stimulate *UHRF1* expression by directly binding to different sites in its promoter region [37, 57, 70]. Specificity protein 1 (SP1) and Forkhead Box M1 (FOXM1) also potentiate UHRF1 expression in different cancers [71, 72]. Repression of SP1 activity by T3 receptor pathway activation downregulates UHRF1, relieves p21 from UHRF1-mediated silencing and induces cell cycle arrest at G0/G1 phase in liver cancer cells [71]. Similarly, our recent study suggests that activation of highly expressed membrane integrin CD47 in astrocytoma activates NFκB-mediated signaling and UHRF1 expression, which in turn represses p16, thereby strengthening the tumor promoter role of UHRF1 [48]. High UHRF1 levels are also attributed to downregulation of its epigenetic regulator H3K9 methyltransferase (G9a) in various cancers which works along with Yin Yang transcription factor 1 (YY1) as negative upstream regulator of UHRF1 [73].

Besides increased expression of *UHRF1*, increased stability of *UHRF1 mRNA* through down-regulation of regulatory micro RNAs and increased stability of UHRF1 protein also contribute to abnormal high levels of UHRF1 in different cancers (Figure 2) [8, 74–79]. UHRF1 protein levels are controlled in normal cells by coordination of ubiquitinylating and deubiquitinylating enzymes which regulate its proteosomal degradation (Figure 2). SCF^β-TrCP^ E3 ligase or intrinsic activity of UHRF1 RING domain can induce degradation of UHRF1 by ubiquitinylation [26, 65]. Phosphorylation of serine residue at 108 by casein kinase 1δ helps SCF^β-TrCP^ E3 ligase to recognize and ubiquitinate UHRF1 for degradation [65]. On the other hand, UHRF1 is stabilized and recruited to chromatin by its association with deubiquitinating enzyme USP7. M phase specific kinase CDK1-cyclin B which phosphorylates UHRF1 at serine 652 in the interacting region of USP7 can disrupt this association and lead to degradation of UHRF1 [40, 80]. Considering that USP7 is upregulated in many cancers, this might be one of the possible reason for high levels of UHRF1 in cancer cells [81–83]. UHRF1 is also stabilized by its interaction with long noncoding RNA UPAT (UHRF1 Protein Associated Transcript), which promotes colon tumorigenesis through inhibition of UHRF1 degradation [84]. Pharmacological inhibition of heat shock protein (HSP90) also destabilizes UHRF1 and suppress cancer cell proliferation predicting a role of HSP90 in UHRF1 turnover [85]. Altogether these events result in abnormal high level of UHRF1 in cancers which appears now to be exploitable as a biomarker.

We will now review the potential of UHRF1 to fulfil the features of a biomarker in various types of cancer.

## UHRF1 EXPRESSION IN DIFFERENT CANCERS

### UHRF1 in lung cancer

Lung cancer is the most common and fatal among different types of cancers with an average 5-year survival rate of around 15% [86]. According to latest data, over 1.8 million new cases of lung cancer were reported worldwide in 2012, while in the same year the death toll of lung cancer was around 1.59 million [86]. High smoking incidences and late diagnosis of cancer are major factors contributing to its high mortality rate. Various novel proteins are now being investigated, in search of a superior biomarker and among them UHRF1 has shown encouraging results. Immunohistochemistry (IHC) analysis of 322 lung cancer tissues from Japan and 56 samples from US, revealed an overexpression of UHRF1 in all histological types of non-small cell lung cancer (NSCLC) especially in non-adenocarcinomas [87]. Transcript analysis of samples also showed marked increase of UHRF1 mRNA in 70% of lung cancer cases. As enhanced expression significantly correlated with the advanced stages and malignancy of the cancer, authors proposed UHRF1 as a prognostic biomarker for lung cancer [87]. Similarly, a recent study in Taiwan has predicted a six-gene signature including *ABCC4*, *ADRBK2*, *KLHL23*, *PDS5A*, *UHRF1* and *ZNF551* as better prognostic marker in NSCLC for overall survival time and treatment outcome [88].

UHRF1 overexpression was also confirmed in another study including 105 NSCLC tissues (55 adenocarcinomas and 50 squamous cell carcinomas) along with DNMT1, DNMT3A and DNMT3B [89]. This overexpression resulted in silencing of tumor suppressor genes such as *RASSF1* and *p16*, via promoter hypermethylation in 32.4% and 26% of cases, respectively. Accordingly, in a cell model of lung cancer, knockdown of UHRF1 in A549 cells prevented the tumor suppressor genes *RASSF1*, *CYGB*, and *CDH13* promoters from hypermethylation [89].

### UHRF1 in liver cancer

Hepatocellular carcinoma (HCC) is one of the most prevalent cancers with multiple etiological factors and is the second leading cause of cancer related deaths worldwide [86]. So far, many studies have been carried out to understand the complex nature and poor prognosis of this disease but it is still elusive. A recent study reported overexpression of UHRF1 in HCC of various etiologies and described UHRF1 as an oncogene, that drives global DNA hypomethylation by delocalizing DNMT1 [62]. In this study, expression of UHRF1 was assessed in 109 human HCC cases by qPCR and results revealed abnormally high expression of UHRF1 (averagely 2-fold higher than normal) in 95.41% (104/109) of the cases [62]. UHRF1 protein levels in samples were also in accordance with mRNA levels and were found significantly higher in 73% of tumors but were barely detectable in normal tissue samples [62]. Tumors with higher expression of UHRF1 also had poor prognosis with higher recurrence rate, alpha fetoprotein, microvascular invasion and lower survival rate emphasizing the diagnostic and prognostic potential of UHRF1 in HCC [62]. Similarly, high levels of UHRF1 mRNA were reported in 160 HCC patients notably during later stages II & III of cancer [71]. UHRF1 protein level were also significantly upregulated in 75.7% (52 of 70) of samples when analyzed by western blot [71]. Results were further confirmed by immunohistochemistry analysis of 136 HCC tissue samples which showed high expression of UHRF1 in tumor samples, positively correlating with tumor size, fetoprotein levels and HBV infection [71]. The diagnostic and prognostic capacities of UHRF1, as a novel biomarker in HCC, were also highlighted by a study on Chinese population including 68 HCC specimens [90]. In this study, significantly higher levels of UHRF1 were found in HCC samples by HPLC compared with the adjacent non-cancerous tissues. Of note, the levels of UHRF1 correlated with distant metastasis, tumor area and HBV [90]. Furthermore, elevated levels of UHRF1 also predicted poor prognosis as after 5 years of follow up, the survival rate in high UHRF1 expression group was 29.8% as compared with 81% in low UHRF1 expression group [90]. Another group also reported similar findings where UHRF1 mRNA expression was found significantly increased in 67% (54/80, *P* < 0.05) of HCC specimens [91]. Immunohistochemical staining of 102 pairs of HCC samples included in study also revealed significantly higher staining of UHRF1 protein in cancerous tissues (57.8% vs 32.7%) when compared to non-cancerous tissue. Like previous studies, overexpression of UHRF1 positively correlated with tumor size, staging and poor survival rate of patients [92].

On a cellular aspect, knockdown of UHRF1 inhibited the tumor growth *in vivo* and *in vitro* and induced cell cycle arrest at G2/M phase confirming the oncogenic potential of UHRF1. Targeting of UHRF1 also decreased the migration and invasion of cancer cells by hampering endothelial to mesenchymal transition (EMT) as evidenced by up regulation of (EMT opposing) E-cadherin and down regulation of (EMT favoring) β-catenin, vimentin, N-cadherin and snail in UHRF1 knockdown cells [92]. Overexpression of UHRF1 in hepatocellular carcinoma also negatively regulated the levels of tumor suppressive long non-coding RNA maternally expressed gene 3 (MEG3) via promoter hypermethylation which exerts its tumor suppressive role by induction of p53 [54, 93].

### UHRF1 in gastric cancer

Gastric cancer is one of the most fatal cancers among all malignant diseases, and is accounted for approximately 723,000 world-wide deaths each year. Eastern Asian countries like China, Japan, Taiwan and Philippines have higher incidences of gastric cancer as compared with western countries [86]. In 2013, a study reported high levels of UHRF1 in gastric cancers and explored miR-146a/b mediated regulation of UHRF1 as a novel therapeutic approach in preventing metastasis and treating such cancers [74]. Immunohistochemistry staining of 106 gastric tumors revealed higher expression of UHRF1 in cancer tissues compared with adjacent normal tissues, which correlated with poor differentiation, cancer staging, increased lymph node and tissue metastasis [74]. Kaplan-Meier analysis showed that patients with higher expression of UHRF1 had poor prognosis and shorter overall survival time as compared with patients having relatively lower expression of UHRF1, suggesting abnormal high levels of UHRF1 as independent diagnostic and prognostic marker for gastric cancer [74].

At the cellular level, overexpression of UHRF1 was observed in aggressive gastric cancer cell lines (GC9811-P and MKN28M), which has been suggested to enhance the proliferating capacity of these cells [74]. Reduced levels of UHRF1, induced by miR-146a/b, reactivated tumor suppressor genes like *SLIT3*, *CDH4*, and *RUNX3* via promoter hypomethylation [74]. Consistently, with this notion, same authors further explored the prognostic value of UHRF1 expression in a study including 238 gastric cancer patients [52]. Immunohistochemistry labelling for UHRF1 was found positive in 82% of samples and significantly correlated with poor differentiation and metastasis. Indeed, patients with higher expression of UHRF1 had a very low 5-year survival rate of 19% as compared to patients with negative (38%) or low expression of UHRF1 (30%) suggesting UHRF1 as a significant predictor of gastric cancer prognosis [52].

### UHRF1 in colorectal cancer

Epigenetic silencing of tumor suppressor genes via promoter hypermethylation is commonly reported besides the genetic aberrations in colorectal carcinogenesis and many mechanisms have been proposed for this deregulation. UHRF1 overexpression in colorectal cancer has been observed in several studies and is considered to be involved in promoter hypermethylation mediated repression of TSGs [7, 8, 94]. Wang *et al* first reported the overexpression of UHRF1 in colorectal cancer and suggested its use as a biomarker and a possible therapeutic target for diagnosis and treatment of colorectal cancer [45]. The authors observed a significantly increased UHRF1 expression at both transcriptomic and proteomic levels in colon cancer tissues and found positive association of this overexpression with metastasis, poor clinical staging and *p16* silencing [45]. Overexpression of UHRF1 was also observed in LoVo, DLD1, SW480 and SW620 colon cancer cell lines. Inhibition of UHRF1 in these cells led to upregulation of *p16*, decreased proliferation and migration capacity, as well as cell cycle arrest at G0/G1 and apoptosis [45]. Similarly, in colorectal cells, overexpressed UHRF1 negatively regulated peroxisome proliferator-activated receptor gamma *(PPARG)*, through epigenetic-dependent mechanisms [95]. The consequences were increased endothelial to mesenchymal transition (EMT), growth and cell viability. Furthermore, prognostic values were more significant when both UHRF1 overexpression and PPARG down-regulation were taken into account [95]. Another study in which 231 colorectal cancer tissues and 40 adenoma specimens were analyzed for UHRF1 levels reported similar results [96]. Indeed, immunohistochemistry showed high expression of UHRF1 in the nucleus of 65.8% (152/231) colorectal cancer tissues and of 87.5% (35/40) adenoma samples while little or no expression was found in normal colonic mucosa [96]. Expression of UHRF1 positively correlated with the depth of invasion and E2F-1 levels [96]. So far it is not yet clear why UHRF1 is up-regulated in cancer but some interesting leads are emerging. For instance, an inverse relationship between the levels of UHRF1 and the regulatory miRNA-9 has been reported in colorectal cells, for which high levels of UHRF1 are associated with poor survival rate of patients [75].

### UHRF1 in breast cancer

Like for other cancers, many studies have reported the association of UHRF1 with breast cancer which is one of the leading causes of cancer related deaths in women world-wide, killing around 0.5 million women each year [86]. In 2003, we first reported increased expression of UHRF1 in breast cancer tissues and found a relationship between its expression and pathological grade of cancer [57]. Later UHRF1 overexpression in breast cancer patients was reported by cDNA microarray and qRT-PCR [37]. Overexpressed UHRF1 was further confirmed by the immunohistochemical staining and correlated with poor differentiation of tumors [37]. Recently, a study has investigated UHRF1 as a diagnostic and prognostic marker for breast cancer [97]. In this study, 62 tissue samples were analyzed and compared with 24 adjacent non-cancerous tissues. Higher expression of UHRF1 was observed at both mRNA and protein level in cancerous tissues which significantly correlated with stage of disease and c-erb2 status but was independent of age, menopause, estrogen and progesterone receptor levels [97].

The origin of the enhanced UHRF1 expression in breast cancer remains elusive in contrast to the down-stream events. Notably, increased expression of UHRF1 in breast cancers is believed to aggravate the pathogenesis by silencing *BRCA1* and modulating the estrogen receptor-α expression [51, 98]. UHRF1 overexpression also increased the proliferation and migration potential of breast cancer cells as exogenous expression of UHRF1 in MDA-MB-231 breast cancer cells facilitated their passage through the cell cycle by induction of cyclin D1 and prevention of apoptosis [99]. UHRF1 also confers radioresistance to breast cancer cells by promoting the expression of DNA damage repair proteins Lupus Ku autoantigen protein p70 (Ku-70) and Lupus Ku autoantigen protein p80 (Ku-80) repairing the chromosomal aberrations and also by down-regulating the expression of BAX and other pro-apoptotic proteins [100]. Similarly, it has been observed that specific inhibition of UHRF1, by mRNA targeting, decreased the oncogenic capacity in breast cancer cells and increased their sensitivity to chemotherapy [101, 102].

### UHRF1 in gynecological tumors

UHRF1 expression in cervical cancer is also a good indicator for cellular proliferation and malignancy. Notably, an analysis of 99 cervical biopsies showed UHRF1 as a useful biomarker to discriminate low grade intraepithelial lesions from normal tissues with a sensitivity of 71.4% and to discriminate low grade intraepithelial lesions from high grade intraepithelial lesions with a sensitivity of 97.6% [103]. Another study on cervical squamous cell carcinoma (CSCC) also reported high expression of UHRF1 at both mRNA and protein level in 47 samples and found that silencing of UHRF1 in cervical cancer cells inhibited cell proliferation and induced apoptosis [104]. The reasons why UHRF1 is overexpressed in cervical cancer, is still not yet elucidated and again it is rather the downstream events that have been deciphered in cellular models. Indeed, polyphenolic extracts from plant sources were found to downregulate UHRF1 in the cervical cancer HeLa cell line [47]. This in turn upregulated the tumor suppressor gene *p16* and ultimately halted the progression of the cell cycle and induced apoptosis [47]. Moreover, UHRF1 overexpression in HeLa cells was shown to decrease their radio-sensitivity to γ-radiation by increasing the expression of the DNA repair proteins XRCC4, thus, enhancing the capability of these cells to repair the DNA damaged by radiation [105]. It is remarkable to notice that a paradigm is emerging concerning the decreased sensitivity of cancer cells to chemotherapy through control of the DNA repair machinery by UHRF1.

Besides cervical cancer, the diagnostic and prognostic capabilities of UHRF1 as biomarker have also been evaluated in ovarian cancer, which is the major worldwide contributor in gynecological tumors posing serious threat to the life of women. In a study including 80 samples from ovarian cancer tissues, significantly higher expression of UHRF1 was found at both transcriptomic and protein levels in tumors as compared with adjacent normal tissues. Knockdown of UHRF1 in ovarian cancer cells inhibited their proliferation and induced apoptosis, suggesting UHRF1 as a general indicator of malignancy and an attractive therapeutic target for ovarian cancers [106].

### UHRF1 in prostate cancer

Prostate cancer undergoes profound epigenetic modifications via aberrant DNA methylation and histone post-translational modifications resulting in silencing of tumor suppressor genes [107]. Expression analysis by immunohistochemistry in tissue microarrays of 226 prostate tumor samples revealed significant overexpression of UHRF1 in almost half of tissue samples [108]. This overexpression correlated with poor clinical prognosis as patients with high expression of UHRF1 had reduced median survival rates (10.4 years) as compared to patients with low expression of UHRF1 (12.4 years) [108]. Recently Wan *et al* reported similar results after analyzing expression of UHRF1 in 225 prostate cancer specimens [109]. UHRF1 staining was found in 47.1% of specimens which positively correlated with the Gleason score and the pathological stage of the disease [109]. Patients with higher levels of UHRF1 were found to be at higher risk for biochemical recurrence after radical prostatectomy. Mean biochemical recurrence (BCR) free time in UHRF1-positive patients was around 23.0 months versus 38.9 months in UHRF1-negative patients while 5-year BCR-free survival rate was 12.4% in UHRF1-positive patients as compared with 51.8% in UHRF1-negative patients. These results support UHRF1 as a valuable independent prognostic factor to predict prostate cancer outcome after radical prostatectomy [109].

At the cellular level, overexpression of UHRF1 has also been reported in aggressively proliferating, androgen-independent cell lines of prostate cancer (DU145 and PC3), while low expression of UHRF1 was found in immortalized normal prostate epithelial cells (LHS) or androgen-dependent prostate adenocarcinoma cells with low metastatic potential (LNCaP and 22Rv1 cells) [108, 109]. Overexpression of UHRF1 accompanied with downregulation of tumor suppressor genes and increased expression of EZH2 (H3K27 methyltransferase) in prostate cancer cells contributed to the poor clinical prognosis and lethal progression disease. UHRF1 also recruited SUV39H1 (H3K9 methyltransferase) and DNMTs to the promoter region of many tumor suppressor genes *(CDH1, PSP94, RARB)* resulting in increased methylation of histones and DNA with subsequent silencing of TSGs [108]. Altogether these results suggest that UHRF1 may serve as a useful biomarker and therapeutic target for prostate cancer as it plays an important role in epigenetic silencing of TSGs via histone and DNA modifications

### UHRF1 in bladder cancer

UHRF1 has also been described as a ‘novel’ diagnostic and prognostic marker for the bladder cancer, which is the second most common cancer of the urinary system [110]. Expression of UHRF1 was found significantly increased in the cancer cells and was positively correlated with histological and pathological grade, as higher expression was observed in later stages of cancer. Increased expression of UHRF1 was also associated with poor prognosis of disease as patients having higher levels of UHRF1 had poor survival rate and higher recurrence [110]. UHRF1 levels evaluated by qRT-PCR or immunohistochemistry based detection methods in surgical sections showed UHRF1 as a specific and sensitive biomarker for bladder cancer. Significantly higher levels of UHRF1 were detectable in specimens with non-invasive or superficially invasive cancers at very early stages compared to normal cells [110]. Similarly, in non-muscle-invasive bladder cancer (NMIBC) increased expression of UHRF1 was found in cancer cells, which was directly related with tumor malignancy [111]. Indeed, patients with UHRF1 overexpression had shorter survival duration (mean survival time 42.59 months) and higher incidences of recurrence (41 out of 70 cases) as compared with patients with relatively lower expression of UHRF1, who had greater survival time (mean survival time 71.36 months) and lower chances of recurrence (29 out of 70 cases) [111]. This suggests UHRF1 as an independent prognostic marker for the bladder cancers.

Other studies reported similar overexpression of UHRF1 in bladder cancers and in invasive cell lines, such as 253J, T24, KU7, along with silencing of tumor suppressor genes *e.g*., *KISS1* and *RGS2* [56, 112, 113]. Altogether, these studies emphasize UHRF1 as an attractive biomarker and therapeutic target for bladder cancers.

### UHRF1 in renal cancer

Each year 338,000 new cases of kidney cancers, with a majority of renal cell carcinomas (RCC) are reported worldwide with a high prevalence in developed countries [86]. First evidence of UHRF1 overexpression in kidney tumors has been reported by Unoki *et al* [110]. By investigating mRNA levels, UHRF1 overexpression was found to be associated with several characteristics of kidney tumor patients, including 5-year survival rates, pathological staging and histological grade [110]. Later Ma *et al* found elevated levels of UHRF1 mRNA in 70% of RCC cases [114]. Overexpression was further confirmed by staining of UHRF1 in histological samples, which showed 74.2 % positive staining in RCC carcinoma tissues [114]. Similarly, UHRF1 overexpression, in metastatic renal cancer tissues as compared with non-metastatic tissues, correlated with downregulation of non-coding miR-146a-5p, which targets UHRF1 transcription [115]. However, another miRNA might also be involved in UHRF1 overexpression in RCC. Indeed, miRNA-101 has also been shown to regulate UHRF1 expression since its downregulation leads to UHRF1 upregulation [78]. Interestingly, in this study UHRF1 overexpression was confirmed in sunitinib-treated RCC tissues and was associated with shorter overall survival after surgery for RCC [78].

### UHRF1 in other cancers

Few studies have also predicted UHRF1 as a diagnostic and prognostic marker for various other types of cancers. Representational difference analysis (RDA) of different pathological grades of astrocytoma revealed *UHRF1* and four other genes to be differentially expressed in astrocytoma cancer tissues [116]. Results were confirmed by qPCR analysis in which 7 normal brain tissues, 9 grade I (pilocytic astrocytoma), 9 grade II (low grade astrocytoma), 11 grade III (anaplastic astrocytoma), and 22 grade IV (glioblastoma multiforme) samples were analyzed. Significant overexpression of UHRF1 was observed in cancerous tissues as compared with normal cells showing the possibility to use this differential expression of UHRF1 as a diagnostic marker for astrocytoma [116].

The diagnostic and prognostic value of UHRF1 has also been evaluated in medulloblastoma, a common malignant brain tumor. Out of 168 formalin-fixed, paraffin-embedded medulloblastoma, high levels of UHRF1 were found in 108 cases while lower expression of UHRF1 was observed in the remaining 60 samples, whilst normal cerebellum tissue samples lacked UHRF1 staining [117]. Kaplan–Meier survival analysis showed that patients with high levels of UHRF1 had poor overall survival and progression free survival rate illustrating UHRF1 as a potential independent prognostic marker for medulloblastoma [117].

UHRF1 has also been proposed as a biomarker and potential therapeutic target for gallbladder cancer, which is well known for its poor prognosis and high mortality rate [118]. Immunohistochemical results showed UHRF1-positive staining in 63.2% of cancerous tissue samples [118]. UHRF1 was overexpressed in cancerous tissues and correlated with the advanced stage and lymph node metastasis. Enhanced expression of UHRF1 was also observed at both mRNA and protein level in GBC-SD and NOZ cell lines and depletion of UHRF1 by siRNA or shRNA markedly reduced their migration potential *in vitro* and tumor forming capabilities [118]. Interestingly, knockdown of UHRF1 promoted the expression of *promyelocytic leukemia protein* (PML) and *p21* (*CDKN1A)* tumor suppressor genes, resulting in cell cycle arrest at G1 [118]. UHRF1 depletion also induced apoptosis in these cells by activating both intrinsic and extrinsic pathways for apoptosis, in accordance with previous studies suggesting that UHRF1 exhibits anti-apoptotic properties [119]. All this information suggests an oncogenic role of UHRF1 in gallbladder cancer and increased expression of UHRF1 as an independent biomarker for diagnosis and a therapeutic target of gallbladder cancers.

Correlation of UHRF1 expression with tumorigenesis has also been demonstrated in laryngeal squamous cell carcinomas (LSCC), through analysis of 60 LSCC samples [120]. UHRF1 overexpression was found in 78.3% (47/60) of cancer tissue samples, whereas remaining 13 samples had relatively lower expression of UHRF1 and in normal tissues, UHRF1 expression was barely detectable [120]. UHRF1 overexpression also correlated with the histological and pathological stages of cancer and was found in undifferentiated cells in advanced stages of cancer [120].

Similar findings were reported in esophageal squamous cell carcinoma (ESCC) where increased expression of UHRF1 was observed in 67% of human ESCC samples and overexpression positively correlated with advanced pathological and histological stages of the cancer, poor differentiation and lymph node metastasis [121]. Accordingly, overexpressed UHRF1 was also related to the radiotherapy resistance in patients with ESCC. Furthermore, results were validated by lentivirus mediated targeting of UHRF1 by shRNA in a TE-1 cell line inducing radio-sensitivity and apoptosis in ESCC derived cell line [121]. Another cohort study of 160 ESCC patients demonstrated that UHRF1 is as an attractive prognostic marker and potential target for cancer therapy as high levels of UHRF1 corresponded to poor survival rate [122].

High levels of UHRF1 have also been reported in several studies on pancreatic cancer, supporting the use of UHRF1 as a diagnostic marker for pancreatic cancer. For instance, power blot assay identified UHRF1 among differentially expressed proteins in pancreatic adenocarcinoma, which is extremely aggressive and difficult to diagnose with survival rate of less than 5% in five years [123]. Moreover, UHRF1 was selectively overexpressed in pancreatic adenocarcinoma tissues while it was not detectable in normal pancreatic tissue or chronic pancreatitis specimens [123]. UHRF1 overexpression was found at both proteomic and transcriptomic level in 80% of pancreatic ductal adenosarcoma cases and high UHRF1 levels correlated with neoplastic grade and lesion [123]. Similarly, UHRF1 overexpression was observed in 86% (114 of 132) of malignant pancreatic tumors samples [124] and 158 pancreatic cancer samples [125]. Furthermore, high UHRF1 levels positively correlated with short survival time of patients [124, 125]. All these results suggest UHRF1 as a valuable independent diagnostic marker for pancreatic cancer in clinical settings.

Similar findings were reported in thyroid cancers cells as microarray analysis showed significant upregulation of UHRF1 to identify gene expression profile that favors the progression of well differentiated tumors to aggressive, poorly differentiated or undifferentiated cancer cells [126]. UHRF1 levels were significantly higher in both differentiated and poorly differentiated cancer cells as compared with normal cells, suggesting a good diagnostic value for UHRF1 in thyroid cancers [126]. These results were in agreement with another study in a Chinese population showing high expression of UHRF1 in poorly differentiated anaplastic thyroid cancer cells versus papillary thyroid cancer and normal cells [127]. Targeting UHRF1 in these cells resulted in suppression of dedifferentiation and stem cell marker expression such as CD97, SOX2, OCT4 and NANOG, highlighting UHRF1 as an attractive target for thyroid cancer therapy [127].

## CONCLUSION AND FUTURE DIRECTIONS

UHRF1 overexpression is found in majority, if not all, of cancers, thus predicting UHRF1 as an independent universal diagnostic and prognostic biomarker for cancer detection, disease progression and therapeutic response monitoring (Table 1). High UHRF1 mRNA and protein levels are detected in early stages of many tumors suggesting UHRF1 as a valuable diagnostic marker for the timely detection of cancers. It is also employed to predict the prognosis of cancer as high level of UHRF1 is generally correlated to poor survival rate, resistance to therapy and recurrence of malignancy.

**Table 1:** Summary of studies describing diagnostic and prognostic potential of UHRF1 in various cancers

Cancer	Methods	Potential of UHRF1	Downregulated TSGs	Reference
Lung Cancer	qRT-PCR, IHC	UHRF1 overexpression relates to tumor stages, metastasis and poor prognosis.	*RASSF1, p16, CYGB* *CDH13*	[87–89]
Liver Cancer	qRT-PCR, IHC, Immunoblot assay, HPLC	UHRF1 overexpression relates to tumor size, metastasis, α-fetoprotein, relapse and short survival time.	*p21, CDH1, MEG3*	[54, 62, 71, 90–92]
Gastric Cancer	qRT-PCR, IHC	UHRF1 overexpression relates to poor differentiation, tumor stages, metastasis and low survival rate.	*SLIT3, CDH4, RUNX3, p16, FOXO4, PPARG, BRCA1, PML*	[52, 74]
Colorectal Cancer	qRT-PCR, IHC	UHRF1 overexpression relates to metastasis, tumor stage, E2F1 levels and poor survival rate.	*p16, PPARG*	[45, 75, 95, 96]
Breast Cancer	qPCR, Western Blot, IHC	UHRF1 overexpression relates to tumor stages, low survival rate and resistance to radiotherapy.	*BRCA1*	[37, 51, 97, 100]
Cervical Cancer	qRT-PCR, Western Blot, IHC	UHRF1 overexpression relates to tumor stages, poor prognosis and resistance to radiotherapy.	*p16*	[47, 103–105]
Ovarian Cancer	qRT-PCR, Western Blot	UHRF1 overexpression relates to progression of cancer.		[106]
Prostate Cancer	qRT-PCR IHC	UHRF1 overexpression relates to high Gleason score, tumor stages, recurrence and low survival rate.	*CDH1, PSP94, RARB*	[107–109]
Bladder Cancer	qRT-PCR, IHC	UHRF1 overexpression relates to tumor stages, risk of recurrence and low survival rate.	*KISS1, RGS2*	[56, 76, 77, 110–113]
Renal Cell Carcinoma	qRT-PCR, Western Blot, IHC	UHRF1 overexpression relates to tumor stages of cancer, drug (sunitinib) resistance and low survival rate	*p53*	[78, 114, 115]
Astrocytoma	RDA, qRT-PCR	UHRF1 overexpression relates to stages of cancer.		[116]
Medulloblastoma	IHC	UHRF1 overexpression relates to shorter survival and progression free time.		[117]
Gall Bladder Carcinoma	qRT-PCR, Western Blot, IHC	UHRF1 overexpression relates to tumor stages and lymph node metastasis.	*PML, p21*	[118]
Laryngeal Squamous Cell Carcinoma	qRT-PCR, IHC	UHRF1 overexpression relates to tumor stages, metastasis and low survival rate.		[120]
Esophageal Squamous Cell Carcinoma	qRT-PCR, IHC	UHRF1 overexpression relates to poor differentiation, pathological stage, low survival rate and resistance to radiotherapy.		[121, 122]
Pancreatic Carcinoma	qRT-PCR, IHC	UHRF1 overexpression relates to tumor size, metastasis, stages of cancer and low survival rate.	*RASSF1, p16, KEAP1*	[123–125]
Thyroid Cancer	qRT-PCR, IHC	UHRF1 overexpression relates to tumor stage.		[126, 127]

Abbreviations: qRT-PCR: quantitative reverse-transcriptase polymerase chain reaction; IHC: immunohistochemistry; RDA: representational difference analysis.

UHRF1 levels have been well correlated with Ki67 and PCNA which are widely used proliferation markers in cancers [52, 95, 104]. However, UHRF1 overexpression is a better diagnosis and prognostic biomarker in cancers as compared with Ki67 and PCNA since it fulfills the requirement of an independent factor. However, so far no universal biomarker is available for cancer early-onset diagnostic. Ratio of Ki67-staining vs UHRF1-staining might differentiate well between normal proliferating cells and cancer cells. Indeed, overexpression of UHRF1 is maintained throughout the cell cycle in cancer cells but not in normal cells [57]. Thus, one might expect that UHRF1-staining should be lower than Ki67 in normal tissues and as much as Ki67 or above in cancer cells. This interesting direction requires further investigations but may represent the basis for the development of a diagnostic kit.

**Figure 3:**
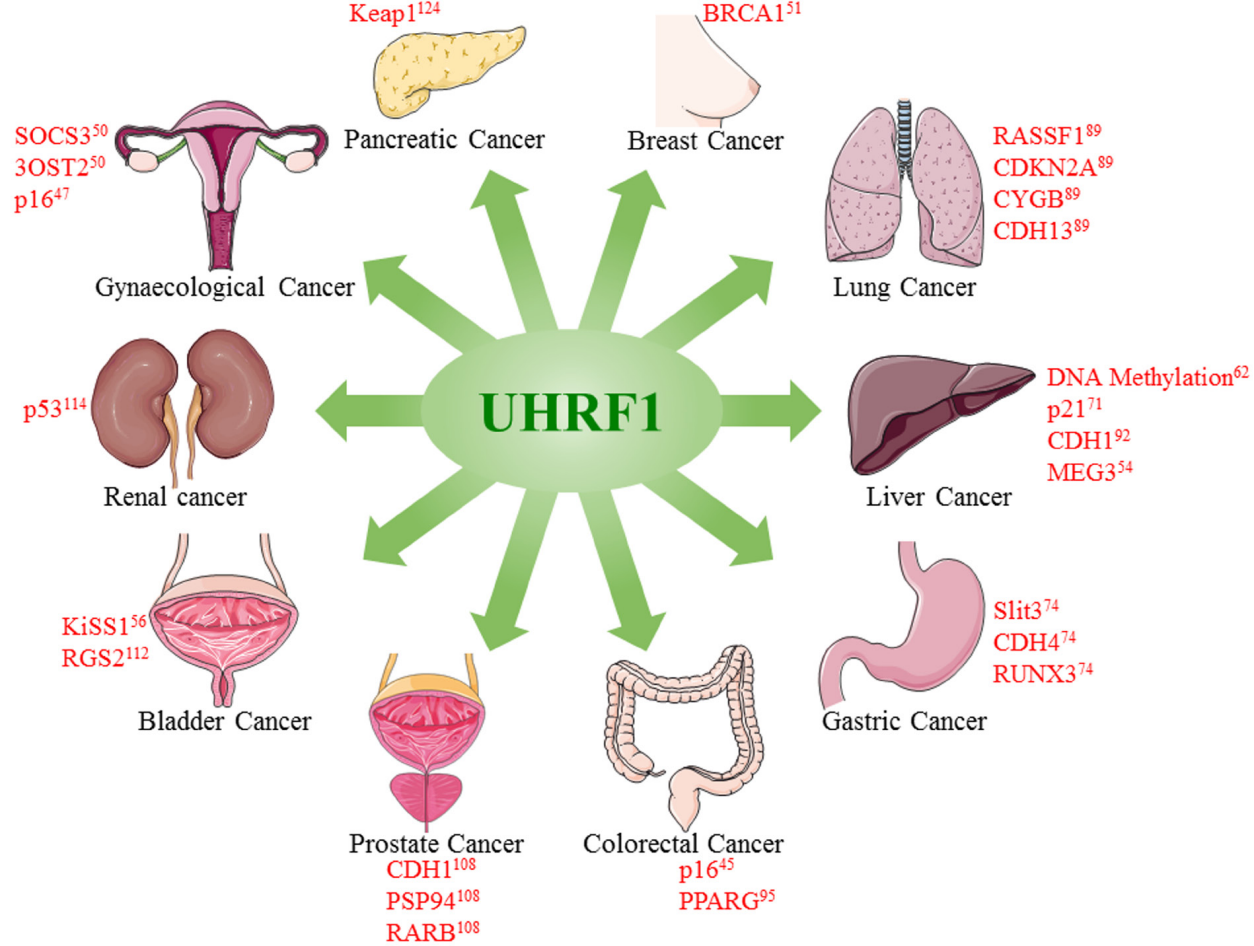
Overexpression of UHRF1 promotes tumorigenesis in different cancers UHRF1 overexpression leads to epigenetic abnormalities including DNA methylation and downregulation of tumor suppressor genes or lnRNAs. Figure is made using images taken with permission from Servier Medical Arts http://servier.com/Powerpoint-image-bank.

UHRF1 overexpression has also proven to be a barrier to cure cancer because of its ability to silence tumor suppressor genes depending on the cancer type (Figure 3) or to counteract pro-apoptotic genes and to induce therapy resistance. It is therefore essential to target UHRF1 overexpression to achieve therapeutic goals in cancer patients. Many strategies can be designed to target UHRF1, including use of small molecules [128]. Therefore, following UHRF1 levels in fluids or tissues during cancer treatment could be of help in a theranostic context.

## Abbreviations

UHRF1: ubiquitin-like containing PHD and Ring Finger domain protein 1
DNMT1: DNA methyltransferase 1
qRT-PCR: quantitative reverse-transcriptase polymerase chain reaction
IHC: immunohistochemistry
RDA: representational difference analysis
NSCLC: non-small cell lung cancer
HCC: hepatocellular carcinoma
NMIBC: non-muscle-invasive bladder cancer
RCC: renal cell carcinoma
ESCC: esophageal squamous cell carcinoma
LSCC: laryngeal squamous cell carcinomas.
